# Non-Typhoidal *Salmonella* as an Unusual Cause of Deep Neck Abscess

**DOI:** 10.1590/0037-8682-0339-2025

**Published:** 2026-02-09

**Authors:** Hilal Kırmızıgül, M. Alperen Kılıç, Emre Emekli, Murat Tepe

**Affiliations:** 1 Eskişehir Osmangazi Üniversitesi Tıp Fakültesi Hastanesi, Eskişehir, Turkey.

**FIGURE 1:**
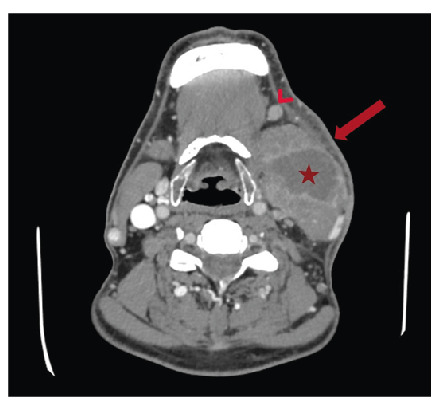
Loculated fluid collection with a thick, contrast-enhancing wall, consistent with an abscess, is present in the left submandibular region (asterisk). Adjacent skin and subcutaneous tissues exhibit thickening and increased reticular density caused by edema (arrow). A reactive lymph node of pathological size is noted next to the abscess (arrowhead).

**FIGURE 2:**
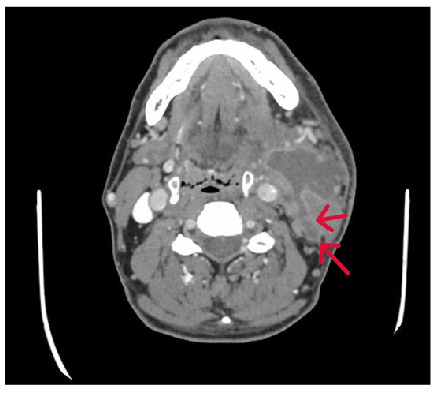
A blind-ending fistulized component of the abscess extending into the posterior cervical region (arrow).

A 52-year-old man presented to the emergency department with a 15-day history of worsening swelling, pain, and erythema on the left side of his neck. Contrast-enhanced computed tomography (CECT) revealed thick-walled, rim-enhancing, loculated fluid collection in the left submandibular space, mainly affecting the sternocleidomastoid (SCM) muscle and extending backward into the posterior cervical space (Figure 1).A blind-ending fistulous component indicative of an abscess was identified (Figure 2).

**FIGURE 3:**
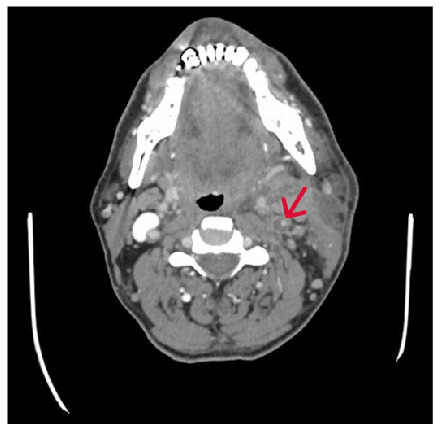
Significant narrowing observed at the origin of the left internal carotid artery (arrow).

Additional imaging findings included thickening of the skin and subcutaneous tissue, increased reticular density attributable to edema, multiple enlarged reactive lymph nodes, and significant narrowing at the origin of the left internal carotid artery (Figure 3). This narrowing was interpreted as secondary arteritis rather than atherosclerotic disease in the context of the acute adjacent infection and surrounding inflammatory changes. Laboratory evaluations revealed leukocytosis and elevated C-reactive protein levels. Histopathology confirmed the presence of an abscess, and non-typhoidal *Salmonella* species were isolated from microbiological cultures.

Deep neck abscesses are infections of potential fascial spaces in the neck that often present diagnostic challenges owing to their variable clinical manifestations^1^. Imaging plays a crucial role in identifying drainable collections, establishing differential diagnoses, and detecting complications, such as venous thrombosis, airway compromise, and mediastinal extension^2^. 

Despite medical advances,deep neck abscesses continue to pose high risks of morbidity and mortality due to severe complications, such as sepsis, airway obstruction, descending mediastinitis, and carotid artery involvement. Prompt surgical drainage, targeted antimicrobial therapy, and early detection of life-threatening sequelae are essential for optimal clinical outcomes^3^.
